# P-1775. Descriptive Analysis of Positive Strongyloides Serology Across Three Academic Medical Centers

**DOI:** 10.1093/ofid/ofaf695.1945

**Published:** 2026-01-11

**Authors:** Sandhya Nagarakanti, Jamilah Shubeilat, Ali Abdulsahib, Natasha Dyal, Matt Biondi, Joseph Hentz, Erin Kaleta, Holenarasipur R Vikram

**Affiliations:** Mayo Clinic Arizona, Phoenix, AZ; Mayo Clinic Arizona, Phoenix, AZ; Mayo Clinic Arizona, Phoenix, AZ; Mayo Clinic, Scottsdale, Arizona; Mayo Clinic Arizona, Phoenix, AZ; Mayo Clinic Arizona, Phoenix, AZ; Mayo Clinic Arizona, Phoenix, AZ; Mayo Clinic, Scottsdale, Arizona

## Abstract

**Background:**

*Strongyloides stercoralis* is an intestinal parasitic nematode with a 2% - 7% prevalence in the United States. Immunosuppression can predispose to disseminated infection, concurrent bacterial sepsis and significant morbidity and mortality. We sought to review positive *Strongyloides* serology (PSS) results across 3 academic medical centers.

**Methods:**

Electronic medical records of patients with PSS were reviewed between 2012 and 2023. Extracted data included demographics, country of origin, travel history, testing indication, clinical presentation, treatment regimen and duration. Patients were classified into chronic infection (PSS without additional clinical manifestations), hyper infection syndrome (cutaneous, gastrointestinal tract, or lung involvement), and disseminated infection (evidence of involvement of distant sites beyond hyper infection such as the brain, liver, kidneys, or bloodstream).

**Results:**

Overall PSS rate across the 3 sites was 5.5%. A total of 412 patients with PSS were identified with a mean age of 56.7 years (SD ±14.2); 272 (66%) were male. The primary indication for testing was transplant evaluation in 230 (56%), eosinophilia workup in 84 (20%), suspicion of active infection in 74 (18%), travel history in 27 (7%), and pre-immunosuppression screening in 27 (7%). 401 (97.3%) were categorized as chronic infection,10 (2%) as hyper infection, and one patient (0.2%) as disseminated infection. Treatment was administered to 366 (89.3%) patients: 226 (61.7%) received 2 doses of Ivermectin on consecutive days, 94 (25.7%) received a single dose, 26 (7.1%) received 2 doses repeated 2 weeks later, and 20 (5.5%) received alternative regimens. The patient with disseminated infection received 2 weeks of Ivermectin followed by monthly dosing.

**Conclusion:**

Majority of the patients with PSS were asymptomatic, and treatment regimens were not uniform. Screening should be considered for patients currently receiving or initiating long-term immunosuppression that can potentiate *Strongyloides* dissemination. Developing a targeted testing algorithm based on epidemiologic factors, clinical suspicion, laboratory parameters and nature of immunosuppression can help identify at-risk patients who can receive pre-emptive therapy if infected.

**Disclosures:**

All Authors: No reported disclosures

